# Physiological Measurements and Transcriptomics Reveal the Fitness Costs of *Monochamus saltuarius* to *Bursaphelenchus xylophilus*

**DOI:** 10.3390/ijms25094906

**Published:** 2024-04-30

**Authors:** Jiaxing Li, Ningning Fu, Sixun Ge, Lili Ren, Youqing Luo

**Affiliations:** 1 Beijing Key Laboratory for Forest Pest Control, Beijing Forestry University, Beijing 100083, China; lijiaxing@bjfu.edu.cn (J.L.); gesixun@bjfu.edu.cn (S.G.); 2 Department of Forest Protection, College of Forestry, Hebei Agricultural University, Baoding 071033, China; ningn_fu@hebau.edu.cn

**Keywords:** *Monochamus saltuarius*, *Bursaphelenchus xylophilus*, transcriptome, phoresy, fitness costs, fatty acid metabolism

## Abstract

The pine wood nematode (PWN) uses several *Monochamus* species as vehicles, through a temporary hitchhiking process known as phoresy, enabling it to access new host plant resources. *Monochamus saltuarius* acts as a new and major vector of the PWN in Northeastern China, showing lower PWN carrying capacity and a shorter transmission cycle compared to established vectors. The apparently altered symbiotic relationship offers an interesting area for researching the costs and adaptions involved in nematode–beetle, a specialized phoresy. We analyzed the response and fitness costs of *M. saltuarius* through physiological measurements and transcriptomics. The PWN exerted adverse repercussions on the growth and development of *M. saltuarius*. The PWN accelerated larval development into pupae, while beetle adults carrying the PWN exhibited an elevated abnormality rate and mortality, and reduced starvation resistance. During the pupal stage, the expression of growth-related genes, including ecdysone-inducible genes (*E74EA*), cuticle proteins, and chitin genes (*CHTs*), markedly increased. Meanwhile, the induced immune response, mainly by the IMD and Toll signaling pathways, could be a contributing factor to adult abnormality and mortality. Adult gonads and trachea exhibited enrichment in pathways related to fatty acid elongation, biosynthesis, and metabolism. *FASN*, *ELOVL*, and *SCD* possibly contributed to resistance against PWN. Our research indicated that phoretic interactions between vector beetles and PWN vary throughout the vector’s lifespan, particularly before and after entry into the trachea. This study highlighted the fitness costs of immunity and metabolism on the vector beetle, indicating the adaptation mechanisms and evolutionary trade-offs to PWN.

## 1. Introduction

Pine wilt disease (PWD), caused by the pine wood nematode (PWN, *Bursaphelenchus xylophilus* Steiner & Buhrer, 1934), poses a significant threat to coniferous forests globally [1]. The disease has predominantly affected Asia and Europe, including countries like Japan, China, the Republic of Korea, Portugal, and Spain [2,3,4,5,6]. In China (hereafter referred to as the Chinese mainland), the PWN has broken earlier predictions regarding suitable habitats. The nematode has extended its range from the natural warm temperate zone to the cooler middle temperate zone in Northeast China, with a notable trend of spreading in both the Northeast and Northwest regions of the country [7,8,9,10]. Concurrently, the new host tree genus *Larix* and the vector insect *Monochamus saltuarius* in northeast China have posed escalating threats [9,11,12].

The PWD system is intricately associated with altitude, vector insects, and host species. In previous studies, the PWNs are predominantly found below 1000 m [13]. Their spread relies on the vector beetles. To date, all confirmed vector insects belong to the genus *Monochamus* [14]. Specifically, in North America, the vector insects include *M. carolinensis, M. clamator, M. scutellatus, M. titillator, M. obtusus, M. notatus, M. marmorator*, and *M. mutator*; in Europe, it is *M. galloprovincialis*; and in Asia, it comprises *M. alternatus, M. grandis, M. saltuarius*, and *M. nitens* [11,14,15]. 

The PWN exhibits a high affinity for attaching to the trachea of *Monochamus* species, engaging in a transient symbiotic relationship known as phoresy, facilitating the dissemination of these minuscule nematodes to new host trees. Phoresy is characterized as an inter-organismal interaction in which one organism (traveler) utilizes another organism (vector) to exit an unfavorable environment, promoting its survival and that of its offspring [16,17]. The traveler may dwell within the vector (endo phoresy), which can exert positive effects (e.g., the elimination of parasites or pathogens) or negative effects (e.g., reduced dispersal due to increased burden) directly or indirectly impacting the fitness of their vectors [18,19]. Research on the interactions between vector beetles and PWN have shown that the presence of the PWN affects the behavioral and physiological traits of vector beetles, including altered metamorphic duration, reproductive capabilities, tracheal wall flexibility, flight range, longevity, and so on [20,21,22,23,24]. These results imply an adaptive evolution of PWNs, vector beetles, and host organisms concerning their developmental processes and immune responses. The adaptability to the PWN varied among different vector beetles. For instance, the PWN could boost fecundity in *M. alternatus* [23], whereas Akbulut and Linit observed a detrimental influence on the longevity and reproductive potential of *M. carolinensis* [22]. The loading capacity of the two beetle species against the PWN differed significantly. An individual adult *M. carolinensis* commonly carries up to 20,000 fourth-stage dispersal juveniles (J_Ⅳ_ PWN) upon emergence, while a single adult *M. alternatus* can harbor over 200,000 J_Ⅳ_ PWN [20,24]. The different carrying capacity of the of the PWN may arise from varying levels of suitability for the nematode. If hitchhiking behavior hinders the efficacy of the vector or includes individuals with low fitness, how can this situation be stabilized? Especially in the prolonged PWN infection cycle, could the stability of hitchhiking and carrying capacity relate to the adaptation costs of various species to the PWN?

In China, the identified vectors are *M. alternatus* and *M. saltuarius*. *M. alternatus* predominantly acts as a vector in southern China, exhibiting a wide distribution range extending to the Jilin and Shaanxi provinces in the north and west [25]. It demonstrates excellent carrying and transmission capabilities, with its molecular mechanisms extensively explored. Research indicated that third-stage dispersal juveniles (J_III_ PWN) secreted ascarosides that promoted *M. alternatus* pupation by inducing ecdysone hormone and up-regulated ecdysone-dependent genes expression [23]. Following PWN penetration into the trachea, hypoxia induced an up-regulation of Muc91C protein, which enhanced tracheal elasticity and activated the Toll-like signaling pathway to mitigate oxidative stress [24,26]. Furthermore, the interaction between *M. alternatus* and the PWN might also be modulated by lipid and energy metabolism [27]. 

In comparison to *M. alternatus*, *M. saltuarius* exhibits a lower carrying capacity, a shorter transmission cycle, and a smaller transmission range [20,28,29,30]. In China, the average natural PWN carrying capacity of *M. alternatus* was 3500 [31], while that of *M. saltuarius* was 500 [30]. Yet, a small number of PWN loading also serves as the primary vector for PWN in northeast China, particularly in Liaoning and Jilin provinces. Thus, the variances in the ability of *M. saltuarius* to transport and disseminate the PWN present a valuable model for investigating potential adaptive costs. It is unclear whether a mechanism exists for regulating nematode density on *M. saltuarius*, and if not, whether inducing a fitness cost is necessary to sustain a stable phoretic relationship.

The main objective of this study was to investigate the physiological and molecular dynamic responses of PWN interactions with a new local vector of PWN in northern China. We used the method of the artificial inoculation of PWNs to evaluate the effects of PWNs on the development time, starvation survival time, hemolymph growth hormones concentration, and immune metabolic signal. We studied the molecular dynamic processes and metabolic pathways of *M. saltuarius* in different periods and tissues. This study explored the adaptive mechanism of *M. saltuarius* to the PWN, providing new insights and references for the study of how to maintain the mutually beneficial relationship stably.

## 2. Results

### 2.1. Statistics on the Number of PWNs Carried by M. saltuarius

To determine the optimal developmental stage for laboratory inoculation and to identify the stage with the highest PWN carrying capacity, we compared the amount of PWN carried by laboratory-inoculated *M. saltuarius* at different days post eclosion (dpe) with those collected from the field (Table S1). A total of 126 laboratory-inoculated beetles were evaluated, of which 118 carried PWN, resulting in a carrier rate of 93.65%. In contrast, only 29 out of 82 field-collected beetles were found to carry PWN, a carrier rate of 35.37%. The maximum PWN carrying capacity in laboratory-inoculated individuals was 6960, with an average carrying capacity of 865 ± 124.74. In contrast, the maximum carrying capacity in field-collected individuals was 2930, with an average carrying capacity of 452 ± 136.97. We observed no significant difference in carrying capacity between male and female adult beetles, and both reached their maximum carrying capacity at five dpe (Figure S1). These results suggested that laboratory inoculation was an effective method for carrying high rates of the PWN, and that adults at five dpe may be the most susceptible to the PWN. Therefore, we selected the females at five dpe as one of the PWN group treatment samples for transcriptome and subsequent analysis. 

### 2.2. Effects of the PWN on the Development and Starvation Resistance of M. saltuarius

**Figure 1:**
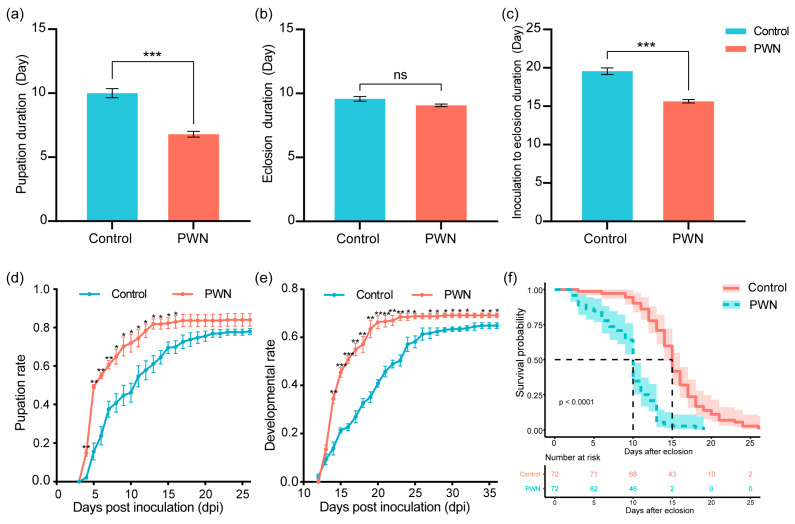
Effects of the PWN on the development and starvation survival time of *M. saltuarius*. (**a**) The effect of the PWN on the duration from inoculation to pupation. (**b**) The effect of the PWN on the eclosion duration. (**c**) The effect of the PWN on the duration from inoculation to eclosion. (**d**) The effect of the PWN on the daily pupation rate. (**e**) The effect of the PWN on the daily developmental rate. (**f**) The survival rate of beetles after PWN treatment. Values were expressed as mean ± SEM. * *p* < 0.05 versus control, ** *p* < 0.01 versus control, and *** *p* < 0.001 versus control.

We evaluated the effects of the PWN on the developmental duration of *M. saltuarius*, including the specific duration from inoculation to pupation, pupal duration, pupation rate, eclosion rate, adult abnormality rate, daily pupation rate, and daily developmental rate. There were no significant differences observed in the pupation rate and eclosion rate between the PWN group and the control group. However, the adult abnormality rate increased significantly (Table 1). The duration from inoculation to pupation in the PWN group was approximately 3 days shorter than that in the control group. Specifically, the duration in the PWN group was 7 ± 0.2 days, while in the control group, it was 10 ± 0.4 days (Figure 1a). There was no significant difference in the pupal duration between the PWN and control groups, which averaged around 9 days (Figure 1b). The development time from larva to adult in the PWN group was shortened by about 4 days (Figure 1c). The fifth instar larvae of the PWN group began to pupate on the four days post inoculation (dpi). From the four dpi to the sixteen dpi, the daily pupation rate in the PWN group was always significantly higher than that in the control group (Figure 1d). Moreover, the daily development rate of the PWN group significantly increased starting from the fourteen dpi (Figure 1e). The findings suggested that the PWN accelerated the growth and development of *M. saltuarius*, while reducing the quality of the adult. 

In addition, starvation resistance was compared in *M. saltuarius* beetles inoculated and not inoculated with the PWN. Every beetle inoculated with the PWN died within twenty dpe, with a median survival of 10 days. Beetles without PWN inoculation died within twenty-seven dpe, with a median survival of 15 days. A Mann–Whitney test revealed that the starvation survival time in the PWN group was significantly different, at 5 days shorter, than that in the control group (*p* < 0.0001) (Figure 1f). These findings suggested that adult starvation resistance in *M. saltuarius* is more vulnerable to PWN.

### 2.3. The PWN Caused a Transcriptomic Shift in the Different Developmental Stages in M. saltuarius

**Figure 2:**
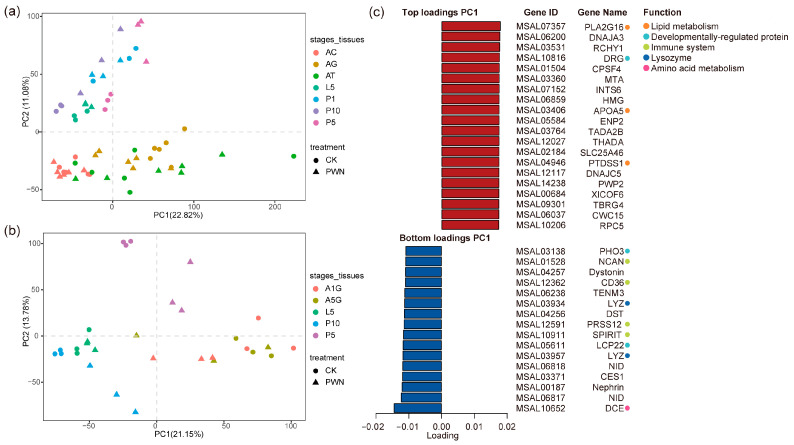
Principal component analysis (PCA) of the transcriptome of *M. saltuarius*. The samples were colored by (**a**) different developmental stages and adult tissues or (**b**) significantly different stages in the transcriptome. (**c**) The top and bottom loadings of the first principal component (PC1) and its annotation to gene function.

Building upon the impact of the PWN on the development and starvation resistance of *M. saltuarius*, we investigated if the PWN could trigger transcriptional physiological responses. According to Figure 2, we categorized the control group and the experimental group into 10 treatment stages. A total of 60 cDNA libraries were constructed. Each sample yielded an average of 4.91 Gb of data (Table S2). The Q30 level ranged from 92.82% to 96.04%. Among the 60 samples, 73.84–89.79% of the clean reads were mapped to the reference genome. High-quality sequencing results were utilized for further analysis.

Principal component analysis (PCA) analysis unveiled distinct patterns contingent upon both developmental stage and tissue response to the PWN in *M. saltuarius*. The first PCA distinctly segregated the transcriptomes based on developmental stages (Figure 2a). Differential responses to PWN were observed between early stage (larvae and pupae) and late stage (adult) specimens. The second principal component highlighted various stages exhibiting notable distinctions (Figure 2b), with some stages showing clear separation along PC1. Pupae P5 and P10 exhibited contrasting reactions to adult A1G and A5G, potentially leading to varied responses in the pupal stage and adult gonads. We examined the genes primarily responsible for the segregation observed on the second PC1 (Figure 2c). Lipid metabolism and growth and development-related genes, including apolipoproteins in phospholipase and PPAR signaling pathways, were linked to positive PC1 values, whereas immunity and lysozyme genes were associated with negative PC1 values. The results showed that *M. saltuarius* may have different response patterns to PWN. The findings indicated that *M. saltuarius* could exhibit diverse response patterns towards PWN. 

A total of 6461 differentially expressed genes (DEGs) were obtained. The majority of DEGs were identified at stages P5 and P10 (3982 and 2673, respectively), with lower numbers found in the A1G (372) and A5G (207) groups (Figure S2). A two-dimensional hierarchical cluster analysis was performed on total DEGs (Figure S3). Four major gene clusters exhibited distinct expression patterns. Cluster 1 comprised 2745 genes predominantly increased in the trachea and gonads of adult specimens, with reduced expression levels in other developmental stages. Cluster 4 showed a significant increase in the expression of 3173 genes during the pupal stages (P1, P5, P10), followed by a decline in expression levels in the adult stages. Cluster 3 comprised 417 genes that exhibited a peak in expression at the early stages (L5, P1). 

Gene Ontology (GO) enrichment analysis was performed to explore their functions (Table S3). Cluster 4 encompassed genes that responded to PWN during the pupal stage and were involved in epithelial cell development, metamorphosis, and larval or pupal morphogenesis. Cluster 1 comprised genes that were up-regulated rapidly upon PWN inoculation in the trachea and gonads, and were involved in responses to the carbohydrate metabolic process, organic cyclic compound catabolic process, mitochondrial inner membrane, and mitochondrial membrane. Cluster 3 was enriched in innate immune activity including the Toll and immune deficiency (IMD) signaling pathways, regulation of melanization defense response, and so on.

### 2.4. Predictive Functional Analysis of the Transcriptome under PWN Treatment

**Figure 3:**
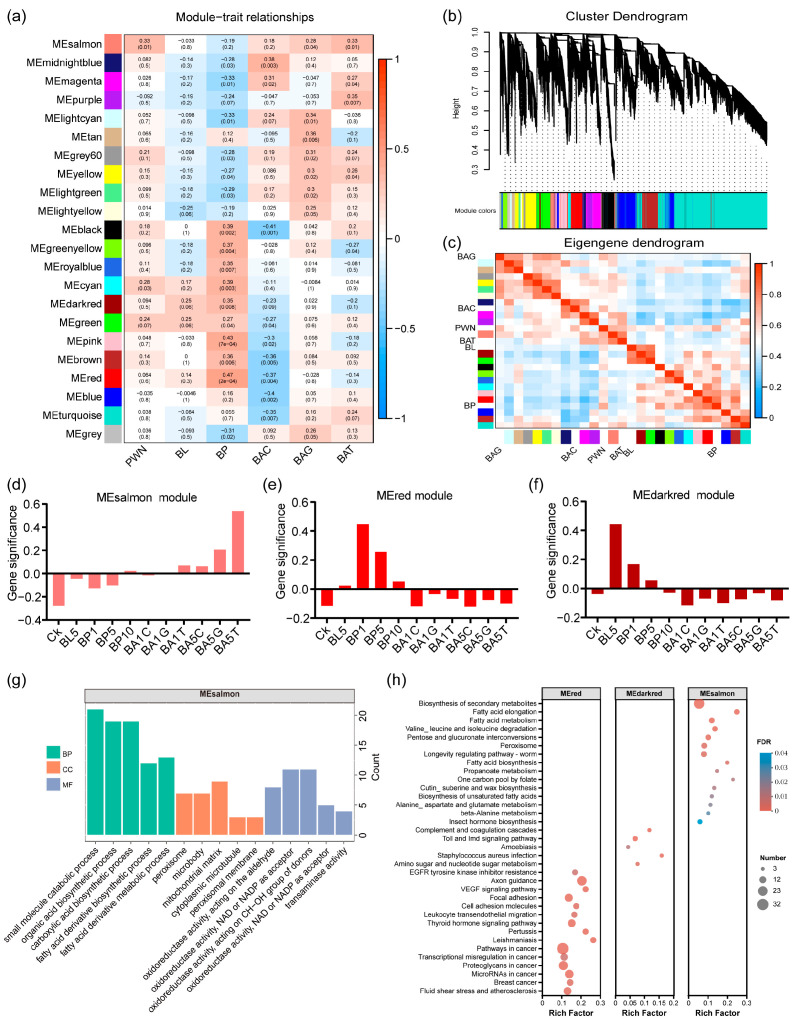
WGCNA analysis and enrichment analysis of *M. saltuarius*. (**a**) Module-trait associations. Each row corresponds to a module. Each column corresponds to a developmental stage. A high degree of correlation coefficient is indicated by dark red or dark blue. The scale on the right represents the correlation range from positive (red, 1) to negative (blue, −1). (**b**) Cluster dendrogram showed 22 co-expression modules identified by WGCNA, which were represented by different colors. (**c**) Hierarchical clustering dendrogram and heatmap of the correlated eigengenes between developmental stages and the PWN. ME, module eigengene. (**d**) The gene expression patterns in hub MEsalmon modules. (**e**) The gene expression patterns in hub MEred modules. (**f**) The gene expression patterns in hub MEdarkred modules. (**g**) GO enrichment analyses of genes involved in MEsalmon module eigengenes. (**h**) KEGG enrichment analyses of genes involved in module eigengenes.

Furthermore, WGCNA analysis was employed to explore the critical stages and tissues, and genes related to PWN response. Twenty-two co-expression modules were identified (Figure 3a,b). Based on Kendall correlation coefficient and *p*-value analysis, seven central modules were identified as most relevant, namely MEsalmon (125 genes), MEdarkred (61 genes), MEgreen (472 genes), MEred (470 genes), MEbrown (759 genes), MEmidnightblue (118 genes), and MElightcyan (101 genes). Cluster analysis revealed the strongest correlation between the MEsalmon module and PWN. Genes within the MEsalmon module exhibited an up-regulated expression pattern during the adult stage (Figure 3c,d). The MEred and MEdarkred modules showed a higher correlation with the pupal stage after PWN treatment, suggesting an up-regulation of gene expression in the pupal stage and a down-regulation in the adult stage (Figure 3e,f). In addition, MEbrown and MEgreen were positively correlated with the larval and pupal stages. Genes in MEmidnightblue were positively correlated with cuticle responses to BAC. MElightcyan participated in the gonad active response to BAG (Figure 3c). MEsalmon were involved in trachea responses to BAT.

Functional enrichment analysis (GO and KEGG) was performed on central modules exhibiting distinct expression patterns (Tables S4 and S5). MEsalmon were mainly enriched in fatty acid metabolism, the peroxisome, insect hormone biosynthesis, and cutin, suberine and wax biosynthesis (Figure 3g,h). MEdrakred and MEgreen were significantly enriched in the Toll and IMD signaling pathways. The MAPK signaling pathway, bacterial invasion of epithelial cells, and other immunity pathways were significantly enriched in MEred and MEbrown (Figure 3h). MElightcyan was significantly enriched for involvement in the lipid metabolism processes, lysosome, and cytochrome P450 (Figure S4). Transcriptome analysis indicated that the PWN had different effects on *M. saltuarius* at different stages and tissues. In the larval and pupal stages, the PWN affected the development and immune response of *M. saltuarius*. In the adult stage, the metabolic process was more active. 

### 2.5. Development and Immune Response of M. saltuarius under PWN Treatment

**Figure 4:**
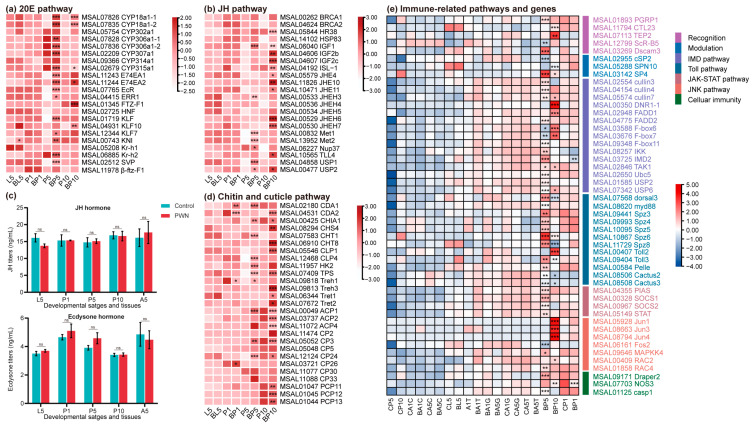
Expression profiles of DEGs and hormone concentration involved in metamorphosis. (**a**) Heatmap of the genes related to ecdysone. (**b**) Heatmap of the genes related to JH. (**c**) Effects of PWN on hemolymph ecdysone and JH concentration in beetles at different developmental stages. Values are expressed as mean ± SEM of three independent experiments. (**d**) Heatmap of the genes related to chitin and cuticle biosynthesis. (**e**) Heatmap of the genes related to immune response related to recognition, signaling pathways, and effectors. * *p* < 0.05 versus control, ** *p* < 0.01 versus control, and *** *p* < 0.001 versus control.

Our study focused on hormone biosynthesis linked to growth and development, along with gene expression involved in cuticle synthesis. We identified 211 DEGs for gene expression analysis (Table S6). Within the ecdysone biosynthesis pathway, pivotal factors for metamorphosis, such as kruppel-like factors (*KLF*) and the early ecdysone-inducible gene (*E74EA*), exhibited up-regulation at the P5 and P10 stages. In the JH biosynthesis pathway, the JH receptor methoprene-tolerant (*MET*) exhibited no significant changes, whereas genes encoding juvenile hormone epoxide hydrolases (*JHEH*) were up-regulated at the L5, P5, and P10 stages. In addition, the hormone inactivation enzyme (*CYP18a1*), and Juvenile hormone degrading enzyme (*JHE*) were also significantly up-regulated at the L5, P1, and P10 stages (Figure 4a). In the cuticle synthesis pathway, cuticle proteins (*CLPs, CPs, PCPs*) and chitin-related genes (*CHTs*) were up-regulated at the P5 and P10 stages (Figure 4d). Expression profiles revealed significant impacts on genes associated with ecdysone synthesis, cuticle protein, and hormone degradation following PWN inoculation.

Alterations in these genes were reflected in hemolymph ecdysone and juvenile hormone (JH) contents. The hormone standard curves are depicted in Figure S5. No significant difference was observed in JH content between the pupal and adult stages in the post-PWN inoculation group. Only the JH content was lower at the L5 stage (Figure 4c), which indicated that the JH hemolymph level was reduced by PWN treatment before pupation. In both larval and pupal stages (P1, P5), the ecdysone content was higher in the PWN group compared to the control group, although the difference was not statistically significant (Figure 4c). PWN boosted the ecdysone hemolymph level in beetle pupae, potentially promoting early pupation. 

WGCNA analysis revealed a negative correlation between immune response and developmental stage. Subsequently, we examined the expression patterns of immune-related genes. We found 289 annotated genes related to immune response, detoxification, and resistance in *M. saltuarius*. These genes included pathogen recognition molecules, signal modulation molecules, intracellular signal transduction molecules, immune response effectors, cellular immune genes, and detoxifying metabolic enzymes (Table S7). In the pupal stage (P5+P10) after PWN inoculation, most immune-related pathways and genes were activated, notably showing a significant up-regulation of the IMD and Toll signaling pathways. Six transcription factors (*FADD, IKK, IMD, cullin, DNR, Ubc,* and *F-box*) involved in the IMD pathway were up-regulated at the P5 and P10 stages (Figure 4e). In the Toll pathway, *Spz, dorsal, Toll2, myd88*, and *cactus* were also up-regulated dramatically. In addition, in the JAK/STAT pathway, the melanization cascade, and recognition factors such as C-type lectin genes (*CTLs*), glutathione transferase (*GST*), and cytochrome P450 (*P450*), were also induced (Figure 4e). 

Conversely, the melanization cascade and most immune-related pathogen recognition molecules, immune response effectors, and signal modulation molecules reverted to baseline expression levels during the adult stage. In adult tissues, genes in the cuticle were less affected. Most genes were down-regulated in the gonad and trachea at the A5 stage. In the IMD pathway, three transcription factors, *USP*, *Cullin*, and *F-box,* were rapidly down-regulated (Figure 4e). Similar expression levels were observed in the JNK and JAK/STAT pathways (Figure 4e). It appeared that the PWN elicited an immune response in the pupal stage, yet this response was suppressed in the adult stage.

### 2.6. PWN Induced Fatty Acid Metabolism in M. saltuarius

We explored the determinants of reduced adult starvation survival time by identifying the central genes within the MEsalmon module. Genes whose weight values were greater than 0.4 were chosen, and their network data were exported to Cytoscape. The top twenty nodes ranked by degree were subsequently chosen (Table S8). Among these, seven genes associated with lipid metabolism (MSAL01140, 00128, 04067, 07179, 08881, 07440, and 07443) were identified, comprising two cytochrome P450 family 4 genes (*CYP4G*), three elongation of very-long-chain fatty acids proteins genes (*ELOVL*), one acyl-CoA desaturase gene (*SCD*), and one fatty acyl-CoA reductase gene (*FAR*) (Figure S6). These genes synergistically catalyze the synthesis of lipid layers and fatty acid derivatives in insects.

**Figure 5:**
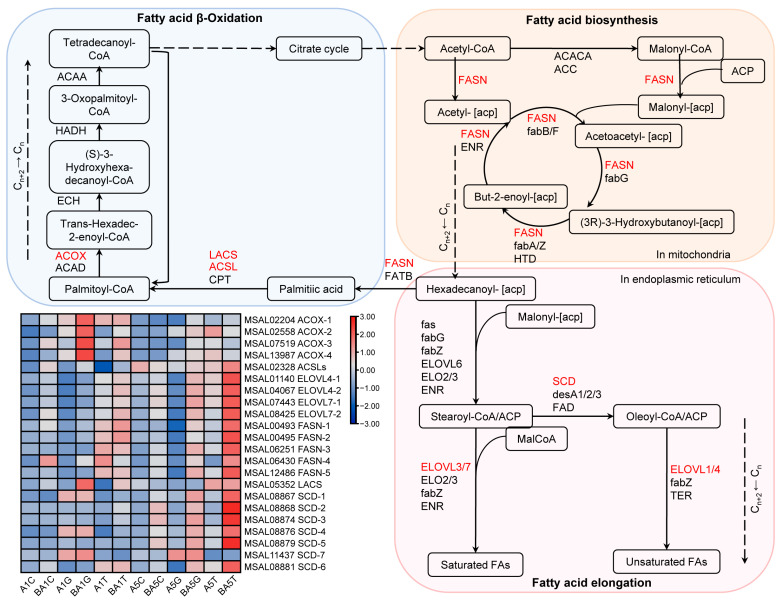
The lipid metabolism of *M. saltuarius* and the heatmap of the DEGs related to these pathways.

To elucidate the impact of the PWN on lipid metabolism, we employed genome sequencing to identify genes associated with fatty acid synthesis, elongation, and oxidation in the transcriptome (Figure 5). We found that the Acyl-CoA oxidase (*ACOX*) genes, related to fatty acid oxidation, were significantly expressed in the gonads of emerging adults. This may be related to the energy requirement of metamorphosis development in the early stages of eclosion. Fatty acid synthetase (*FASN*), involved in fatty acid synthesis, exhibited significant up-regulation in the gonads and trachea, in line with the expression of *ELOVL* and *SCD*, essential for fatty acid elongation (Table S9). *ELOVL* are used to synthesize very-long-chain fatty acids, while desaturase initiates the conversion of monounsaturated fatty acids (MUFAs) to polyunsaturated fatty acids (PUFAs). These findings suggested that FAs, specifically MUFAs or their related genes, may play a critical role in the resistance of *M. saltuarius* to the PWN. PWN may induce lipid metabolism in *M. saltuarius*, which increases energy consumption and reduces the starvation resistance.

### 2.7. Verification of Selected DEGs via RT-qPCR

**Figure 6:**
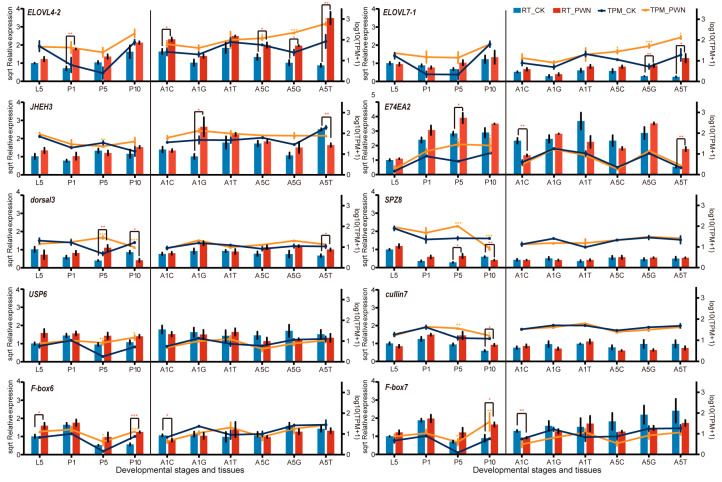
Expression patterns verified by RT-qPCR of development-related genes at different developmental stages and tissues. The RT-qPCR results (columns) are compared with RNA-Seq data (lines). Sqrt relative expression represents the square root of the RT-qPCR value. The normalized expression level (TPM+1) of RNA-Seq is indicated on the y axis to the right. * *p* < 0.05, ** *p* < 0.01, *** *p* < 0.001. The red star symbols indicate significant results from the independent sample t-test of the RT-qPCR values. The orange star symbol signifies the *p* value of differentially expressed genes in the transcriptome.

RT-qPCR was performed to validate the transcriptome analysis. Ten crucial DEGs participating in hormone, immune, and lipid metabolism were selected (Figure 6). *E74EA2* and *JHEH3* were associated with hormone synthesis and played a role in the growth and development of *M. saltuarius*. Compared with the control group, the expression levels of *E74EA2* and *JHEH3* were higher at the L5 stage. There were significant differences at the P5 and P10 stages, which were similar to the transcript results. Gene *dorsal3*, *Spz8*, *USP6*, *Cullin7*, *F-Box6*, and *F-Box7* were associated with immune response. The results showed that genes in IMD (*F-Box6*, *F-Box67*, *USP6*, *Cullin7*) and Toll (*Spz8*, *dorsal3*) pathways maintained high levels at the P5 and P10 stages of beetle inoculation with the PWN. However, they were down-regulated or kept at normal levels at the A1 and A5 stages. These results indicated that the immune response to the PWN was significantly induced at the pupal stage, while most had no significant changes at the adult stage. *ELOVL4-2* and *ELOVL7-1* were key genes involved in fatty acid elongation and synthesis. They were expressed at low levels in the A1 stages and reached a significant expression peak in the A5 stages. In different tissues, the most significant expression was found in the trachea, followed by the gonad. There was no significant difference in the cuticle. 

The expression data and regulation patterns were similar compared with the corresponding values from the RT-qPCR analyses. Generally, the results of selected target DEGs between RNA-Seq and RT-qPCR showed that the data are consistent for relevant genes and pathways. 

## 3. Discussion

Our findings revealed that the adaption of vector beetles was compromised during PWN inoculation and transportation. PWN stress shortened the time for the larval to pupal stages of *M. saltuarius*, inducing the expression of development and immune-related genes. In adults, PWN loading and transmission minimally impacted the longicorn immune response but notably up-regulated fatty acid metabolism in the trachea and gonad. The adult abnormality rate and starvation resistance were also affected. We deduced that prior to PWN introduction, *M. saltuarius* expedited development to resist infestation. During the loading of the PWN, *M. saltuarius* bolstered stress tolerance and reproductive development via fatty acid metabolism. Our study exemplified how vector-induced phenotypic changes under traveler stress in commensal interaction may prompt trade-offs between developmental efficiency and defense mechanisms. 

### 3.1. Effects of the PWN on the Development and Immune Fitness of M. saltuarius

Biological stress can either accelerate or decelerate host development, a phenomenon common in both predatory and parasitic relationships. PWNs initiated pupation in beetle larvae by stimulating the production of ecdysone [23]. Stress from *Harmonia axyridis* shortened the development cycle of *Helicoverpa armigera* and reduced their adult lifespan [32]. The larval stage of *Agalychnis callidryas* was accelerated when stressed by the predatory bug (*Belostoma* spp.), and slowed down when stressed by the foraging aquatic spider (*Thaumasia* spp.) [33]. The choice of strategy by a host is influenced by its ecological traits and depends on which strategy enhances its survival [33,34]. 

This study revealed that PWN stress led to a shortened development period and reduced starvation survival time of *M. saltuarius*. During winter, the larva stops feeding on the host xylem, exhibiting weak activity and metabolism, and hindering defense against external infestation. Upon emergence, it can leave the host, thereby avoiding PWN infestation in the tree by changing its living environment. Notably, prior to infesting longicorn beetles, PWNs were third-stage dispersal juveniles (J_Ⅲ_ PWN), carrying a higher diversity and quantity of microorganisms compared to the J_Ⅳ_ PWN, consequently inducing a more pronounced stress response in the longicorn beetles [35]. Hence, the abbreviated larval metamorphosis period in *M. saltuarius* may be linked to an acceleration strategy. This acceleration facilitates larval pupation, aiding in a quick evasion of microbial stress and enhancing survival rates, with positive implications for population development. 

Such trends were also observed in the *M. alternatus*–PWN interaction model. Zhao et al. suggested that the ascarosides from the PWN elevated ecdysone levels and up-regulated ecdysone-related genes, hastening the pupation of longicorn beetles to synchronize the development [23]. This study quantified ecdysone and JH levels, and assessed the expression of relevant genes across various developmental stages of *M. saltuarius*. Following PWN inoculation, ecdysone increased in both larval and pupal stages. While the ecdysone content of *M. saltuarius* in response to the PWN did not show significant differences, unlike those observed in *M. alternatus*. This variation in response could be explained by their distinct biogeographies and susceptibility to PWN stress. *M. alternatus* is native to the Oriental region, whereas *M. saltuarius* originates from the Palearctic region [36]. Disparities in geographical distribution and evolutionary lineages impact their interaction with the PWN, life cycle, and phenotypic variations [37]. These findings suggested that the PWN could exhibit varying levels of fitness to different vectors and loading capacity.

The KEGG and WGCNA analyses indicated that DEGs in PWN-treated pupae were primarily associated with the Toll immune signaling pathway, detoxification metabolism, and transmembrane transporter activity. The immune response of insects to nematodes is marked by recognition proteins such as *GPRP*, which regulate antimicrobial peptide synthesis via the humoral immune Toll and IMD pathways, in conjunction with the prophenoloxidase-cascade in cellular immunity [38,39,40]. The activation of the Toll pathway in *Drosophila* included the proteolytic cleavage of the ligand *Spz*, resulting in the activation of proteins *Rel, Dif,* and *Dorsal* [41]. In this study, the transcription factors (*Toll, Spz, and dorsal*) of the Toll signaling pathway components were up-regulated on the 5th and 10th day post PWN treatment. Moreover, the recognition factor *PGRP*, and IMD and JNK signaling pathway-related transcription factors exhibited significant up-regulation. Recent research has suggested roles for ecdysone and JH in insect innate immunity, where ecdysone enhanced both cellular and humoral immune responses against foreign pathogens, possibly potentiated by pathogen exposure [42]. In the *Locusta migratoria*, ecdysone could influence the Toll and IMD pathways. The gene expression of immune-related genes such as *PGRP-LE, PGRP-SA, Defensin, Diptericin*, and lysozyme was notably increased upon ecdysone treatment [43]. In *D*. *melanogaster*, AMPs expression was triggered by *EcR* and *USP* [44]. In our study, ecdysone regulatory factors (*BRC, E74, EcR,* and *KLFs*) exhibited up-regulation in both larval and pupal stages, suggesting the PWN’s ability to modulate ecdysone hormone and related synthesis genes. Investigating whether the vector beetle response is influenced by hormone-mediated immunity and development merits additional research.

### 3.2. Activating Fatty Acid Metabolism to Resist the PWN

This study revealed that carrying PWN reduced starvation resistance in *M. saltuarius*, indicating that the PWN influenced lipid metabolism and energy consumption in *M. saltuarius*. Similarly, exposure to mites induced costly defensive behaviors in flies, reduced glycogen and lipid stores, and ultimately shortened fly lifespans as well as lowering fecundity [45,46]. We further observed an up-regulation of metabolic genes expression in *M. saltuarius* in response to PWN attachment and carriage, particularly genes related to fatty acid synthesis and elongation processes in the gonads and trachea. Interactions among species impose selective pressures that alter their metabolomes [47]. For instance, parasitic mites significantly enhanced metabolic and immune responses in *D. melanogaster*. Silencing brummer lipase (*bmm*), a lipid metabolism gene, increased resistance to parasitism carried by mites. Similarly, Trypanosoma rangeli modulated vector *Rhodnius prolixus* lipid metabolism, impacting feeding and behavior. Recent studies have also implicated lipid and fatty acid metabolism in PWN disease systems. Notably, the lipid metabolite C16:1 facilitated successful colonization in the PWN-host-native fungus symbiosis [48]. Ning demonstrated PWN-induced alterations in *M. alternatus* glycolipid metabolism at the epigenetic level, aligning with our findings [27]. The primary physiological cost of low nutrient reserves may stem from combined host defense and starvation resistance responses. 

Fatty acids are essential constituents of lipids, and their functionality relies on the saturation level and carbon chain length. PUFAs serve as vital nutrients for organisms, doubling as an energy reservoir and contributing to the structural integrity of phospholipid membranes, thereby affecting the biophysical properties of the plasma membrane [49]. Very-long-chain fatty acids (VLCFAs) play crucial roles in biofilm and epidermal structures [50]. We found the hub genes, *FAR*, *CYP4G*, *ELOVL*, and *SCD,* involved in lipid synthesis and transport, may play major roles in defense against the PWN. These genes belong to seven protease gene families in the insect lipid synthesis pathway [51]. Inhibiting the *ELOVL* enzyme can impede VLCFA synthesis, leading to enhanced epidermal permeability and facilitating moth adaptation to heat stress [50]. In *Drosophila*, a *FAR* gene has been confirmed as indispensable for tracheal gas exchange. Up-regulating *ELOVL* genes stimulated ultra-long-chain fatty acid production while decreasing membrane permeability [52,53]. *FAS* plays a pivotal role in synthesizing short-chain to medium-chain fatty acids in mitochondria [49]. *ELOVL* elongates long-chain and very-long-chain fatty acid chains in the endoplasmic reticulum [51,54]. The current investigation showcased a significant induction of key genes, *FAS*, *FAR*, *CYP4G*, *ELOVL*, and *SCD,* linked to fatty acid synthesis and transportation in the trachea of *M. saltuarius*. A previous study documented that the PWN induced an up-regulation of a resilin-like mucin protein *Muc91C*, enhancing tracheal elasticity and aECM thickness following the PWN invasion of *M. alternatus* [24]. Our hypothesis posited that *M. saltuarius* may increase phospholipid membrane permeability by boosting fatty acid synthesis, thereby improving elasticity. 

In addition to their role as cell membrane constituents, the synthesis of fatty acids for lipid production serves as a crucial energy source in reproduction and development [55]. Insect oocyte development necessitates rapid lipid absorption, with *Aedes aegypti* oocytes assimilating most proteins and lipids within 2 days post-blood feeding [56,57]. *Serratia symbiotica* enhanced genes connected to fatty acid biosynthesis and the elongation of pea aphids to promote host development [55]. The notable up-regulation of fatty acid biosynthesis and elongation genes in *M. saltuarius* gonads implied a potential role of PWN in promoting reproductive development. Similarly, the numbers of eggs laid by female *M. alternatus* was boosted by PWN asc-∆C6 [23]. The PWN may regulate reproductive development by influencing the lipid metabolism of the beetles. Further investigations are needed to elucidate the interplay between reproductive processes, oviposition, lipid metabolism, and the adaptive strategies of *M. saltuarius* to PWN infestation, potentially affecting survival and reproductive outcomes.

## 4. Materials and Methods

### 4.1. PWN Inoculation and Sample Collection

Samples made up of the 5th instar larvae of *M. saltuarius*, with weights ranging from 300 to 500 mg, were collected from *Pinus koraiensis* in a forest farm around Dahuofang Reservoir in Fushun, Liaoning Province, China in December 2020, 2021, and 2022 for three consecutive years. An artificial co-culture medium of PWN and *M. saltuarius* was prepared following the method described in our previous study [58]. After sterilizing the larvae surface with 75% alcohol, 5th instar larvae were inoculated in sawdust-barley media, inoculating with PWN as treatment, and inoculating without PWN as a control.

Beetle samples were collected from various developmental stages and tissues in the artificial co-culture medium. Samples were collected from larvae on day 5 post inoculation (L5), pupae on days 1, 5, and 10 post pupation (P1, P5, P10), and adults on days 1 and 5 post eclosion (A1, A5). The PWN group (BL5, BP1, BP5, BP10, BA1, and BA5) and the control group (CL5, CP1, CP5, CP10, CA1, and CA5) were recorded, respectively. In addition, based on a previous study on PWN harboring sites [59], the cuticle (A1C and A5C), trachea (A1T and A5T), and gonads (A1G and A5G) of female adults at the A1 and A5 stages were also sampled. The PWN group (BA1C and BA5C; BA1T and BA5T; and BA1G and BA5G) and control group (CA1C and CA5C; CA1T and CA5T; and CA1G and CA5G) were recorded, respectively. The sampling stages and issues were depicted in Figure S7.

Samples of each stage and tissue were divided into three groups. For the first group, the samples were cut into pieces separately, and a petri dish immersion method was used to calculate the nematode populations. For the second group, the hemolymph of samples was extracted to measure ecdysone and JH concentrations. For the third group, after removing the gut, the samples were put into liquid nitrogen immediately and stored at −80 °C until RNA was extracted.

### 4.2. Assays with the Number of PWNs Carried by M. saltuarius

We employed a previously established method [60] to assess the nematode carrying capacity of inoculated beetles. We randomly selected 126 *M. saltuarius* individuals that had emerged on various days in the artificial co-culture medium. The samples were sectioned and transferred to disposable plastic culture dishes with a diameter of 60 mm. Distilled water was added to immerse the beetles completely. Subsequently, the petri dishes were left at room temperature, and after 24 h, we examined and tallied them using an optical microscope. The identical detection approach was applied to field-collected beetles, resulting in a total of 82 field adult beetles identified through the same process.

### 4.3. Development and Starvation Resistance Assays

Beetles inoculated with and without PWNs were cultured independently in dark conditions at 25 °C and 70% humidity. To assess the impact of PWNs on the development of *M. saltuarius*, 282 larvae were inoculated into both the PWN group and the control group, respectively. The number of pupation, eclosion, and dead beetles were monitored and recorded every 24 h for both groups to analyze their growth and development over time. The durations from inoculation to pupation, pupal duration, and eclosion time were documented. Pupation and eclosion rates were calculated as follows: Pupation rate = number of pupae/total larvae. Eclosion rate = number of eclosion events/total pupae [61]. Daily pupation rate = number of pupations per day/total number. Daily developmental rate = number of eclosion per day/total number. To analyze the survival rate of adults, a total of 72 emerging adults were randomly selected from both the PWN and control groups. Their survival status in the medium was monitored every 24 h, and a survival curve was plotted based on the collected data.

### 4.4. Ecdysone and JH Measurement

Samples were obtained from both the PWN group and the control group at different developmental stages (L5, P1, P5, P10, and A5). Beetle specimens were punctured to extract 100 μL of hemolymph, which was then diluted 1:1 with phosphate buffer (1× concentration). The protocols provided with the insect ecdysone ELISA kit (JM-00038O2) and the insect JH ELISA kit (JM-00044O2) from Jingmei Biotechnology Co, Jiangsu, China were followed to quantify the levels of 20E and JH, respectively. Three independent biological replicates were collected from each group, with each replicate comprising a single sample.

### 4.5. RNA Extraction and Transcriptome Sequencing

Total RNA from beetles inoculated with and without PWN was extracted using the EASYSpin Plus Tissue/Cell RNA Kit (Aidlab, Beijing, China) following the manufacturer’s procedure. The quality and quantity of total RNA were evaluated using 1.2% (*w*/*v*) agarose gel electrophoresis and NanoDrop 2000. A total of 60 libraries were constructed. Then, the libraries were sequenced by the Kaitaimingjing Gene Technology Corporation (Beijing, China) using the Illumina HiSeq 4000 platform (Illumina, San Diego, CA, USA). The clean reads were mapped to the reference genome of *M. saltuarius* [62] using the HISAT2 software for subsequent transcript analysis. The raw sequencing data of this study were submitted to the National Center for Biotechnology Information (NCBI) Sequence Read Archive (SRA) databases with the accession number PRJNA907937.

### 4.6. Transcriptome Evaluation and Gene Expression Analysis

Gene expression levels were estimated as transcripts per million reads (TPM) using the RSEM (v 1.3.1) software [63]. Differentially expressed genes (DEGs) between each of the two groups were identified using the DESeq2 R package (v 1.24.0) and are presented as log2 (Fold Change (FC)) values. The *p* values were adjusted using the Benjamini–Hochberg method [64]. PCA analysis was performed with an R package reference to PCAEXPLORER [65]. Genes in the top and bottom loadings of the first PC were extracted and functionally characterized. The R package was used for a hierarchical clustering analysis of the selected DEGs, and genes with similar expression patterns were clustered. A functional enrichment of DEGs was performed by Gene Ontology (GO, http://www.geneontology.org, accessed on 15 June 2023) and the Kyoto Encyclopedia of Genes and Genomes pathway database (KEGG, http://www.genome.jp/kegg/, accessed on 20 June 2023).

### 4.7. Weighted Gene Co-Expression Network Analysis

Weighted gene co-expression network analysis (WGCNA) of the transcript was used to explore the complex relationships between developmental stages, tissues, and PWN inoculation, using an R package (https://github.com/jmzeng1314/my_WGCNA, accessed on 15 April 2023) [66]. After removing genes with very low expressions and low variable coefficients, a total of 10,094 genes were obtained. The Soft Threshold (power) was determined to be 7 when the degree of independence was over 0.8 (Figure S8). The minimum number of genes was set to 30, for high reliability results. Module trait associations were estimated using the correlation between the ME and the trait. Network depictions were constructed using the Cytoscape software (version 3.7.1, https://cytoscape.org/, accessed on 20 April 2023) [67].

### 4.8. Real-Time Quantitative PCR Analysis

Real-time quantitative PCR (RT-qPCR) was used to measure the expression patterns of 10 candidate genes related to growth, immunity, and fatty acid metabolism in *M. saltuarius*. The RT-qPCR was performed with the TB Green^®^ Premix Ex Taq™ II (Takara, Shiga, Japan) and run on the Bio-Rad CFX96 PCR System (Bio-Rad, CA, USA). The gene 40S ribosomal protein S5 (RPS5) was used as the control [58]. Specific primers were designed using the online website, Primer3Plus (http://www.primer3plus.com/cgi-bin/dev/primer3plus.cgi, accessed on 20 July 2023). The primer sequences are listed in Table S10. Three biological replicates and three technical replicates were used for analysis. The relative expression of the genes was calculated using the 2^−∆∆CT^ method [68].

### 4.9. Statistical Analysis

The physiological states, hormone concentration, and starvation resistance of *M. saltuarius* were analyzed by one-way analysis of variance (ANOVA) and non-parametric tests using SPSS 23.0 (IBM SPSS, Armonk, NY, USA) [69]. Multiple tests and corrections were used to calculate the *p* value (*p*), and *p* < 0.05 was considered as statistically significant. Bar charts and line charts were rendered with GraphPad Prism 8 (GraphPad Software, San Diego, CA, USA). Heat maps of gene expression were drawn with R packages and the online website hiplot (https://hiplot-academic.com/basic, accessed on 20 June 2023).

## 5. Conclusions

This study integrated the transcriptome analysis of various development stages and physiological parameters to uncover the adaptive strategies of *M. saltuarius* to the PWN in terms of development, immunity, and metabolism. The substantial responses of *M. saltuarius* to the PWN underscored the metabolic costs for the vector, influencing the balance between developmental survival and immunity. Vector beetles that evaded PWN infestation exhibited heightened gene transcription levels linked to fatty acid metabolism. This implied that fatty acid metabolism may play a crucial role in the adaptation of vector beetles to the PWN, warranting comprehensive and in-depth exploration. These findings have addressed significant gaps in our understanding of the molecular mechanisms involved in the interactions between vector beetles and the PWN, highlighting potential ecological trade-offs in specialized phoresy.

## Figures and Tables

**Table 1:** Effects of the PWN on the developmental duration of *M. saltuarius*.

Treatment	Total	Pupation	Eclosion	Adult Abnormality	Pupation	Eclosion	Adult Abnormality
	Number	Number	Number	Number	Rate (%)	Rate (%)	Rate (%)
PWN	282	238	196	22	84.39 ± 0.03 a	82.57 ± 0.03 a	11.23 ± 0.094 a
Control	282	223	187	12	79.08 ± 0.02 a	83.87 ± 0.02 a	6.40 ± 0.014 b

Note: Each value was the mean ± SEM. In the same column, different letters following the number indicate statistically significant differences by two-way ANOVA (*p* < 0.05).

## Data Availability

All data mentioned in this paper are available at the National Center for Biotechnology Information (NCBI) with the BioProject no. PRJNA907937. Raw sequence data (Illumina, resequencing and RNA-seq) have been deposited in the NCBI Sequence Read Archive (SRA) database at the BioSample no. SAMN32012001-SAMN32012060. We have released our raw data at https://www.ncbi.nlm.nih.gov/bioproject/PRJNA907937, accessed on 14 December 2013.

## Supplementary Materials

The following supporting information can be downloaded at: https://www.mdpi.com/article/10.3390/ijms25094906/s1.
