# Supplementary figures and images for: Physiological Measurements and Transcriptomics Reveal the Fitness Costs of Monochamus saltuarius to Bursaphelenchus xylophilus
Source: Int J Mol Sci. 2024 Apr 30;25(9):4906. doi: 10.3390/ijms25094906 (PMC11084816; doi:10.3390/ijms25094906)

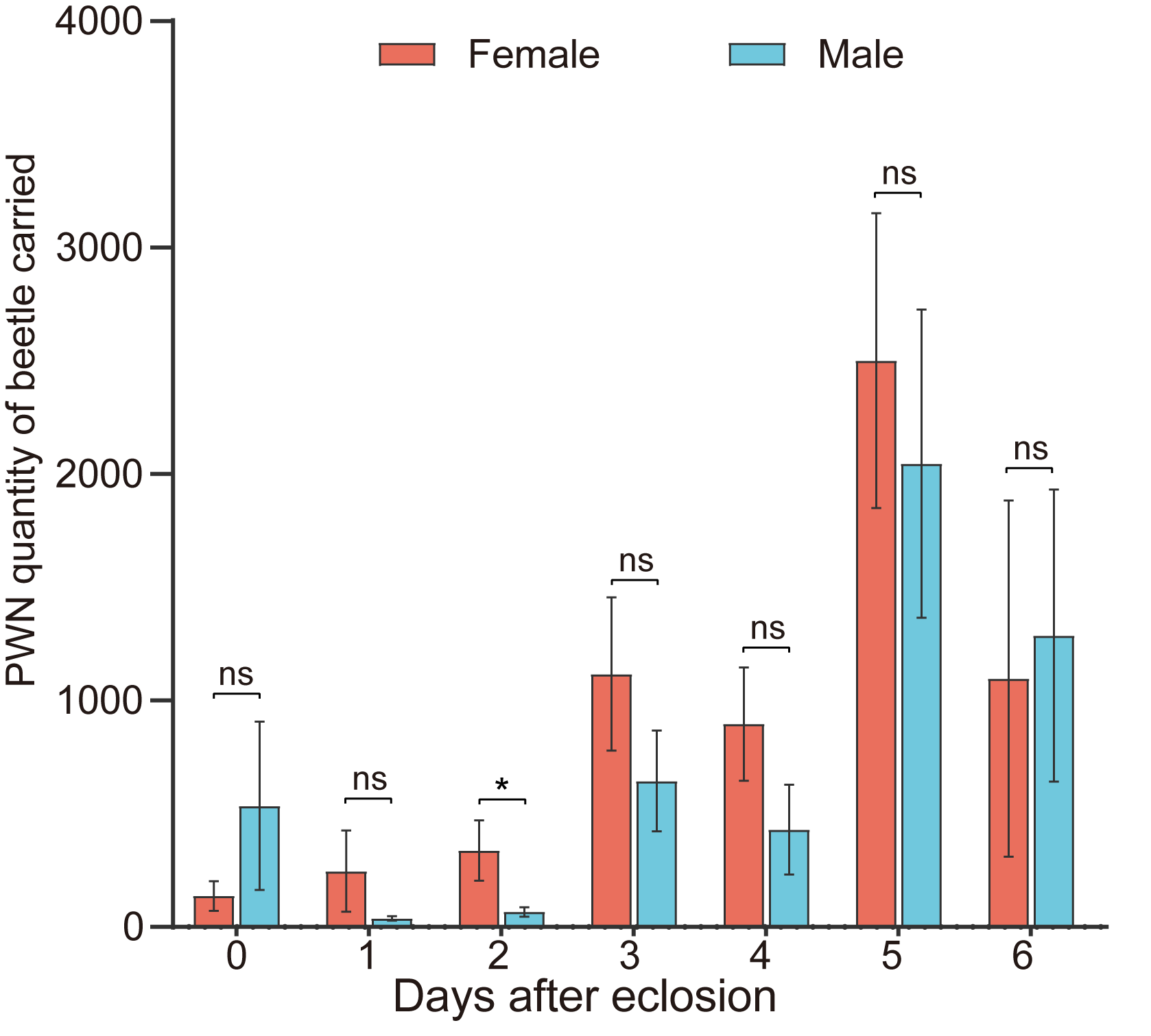

Supplement: Supplementary file 1 [file ijms-25-04906-s001.zip › Supplementary materials/Supplementary figures/Figure S1.tif]

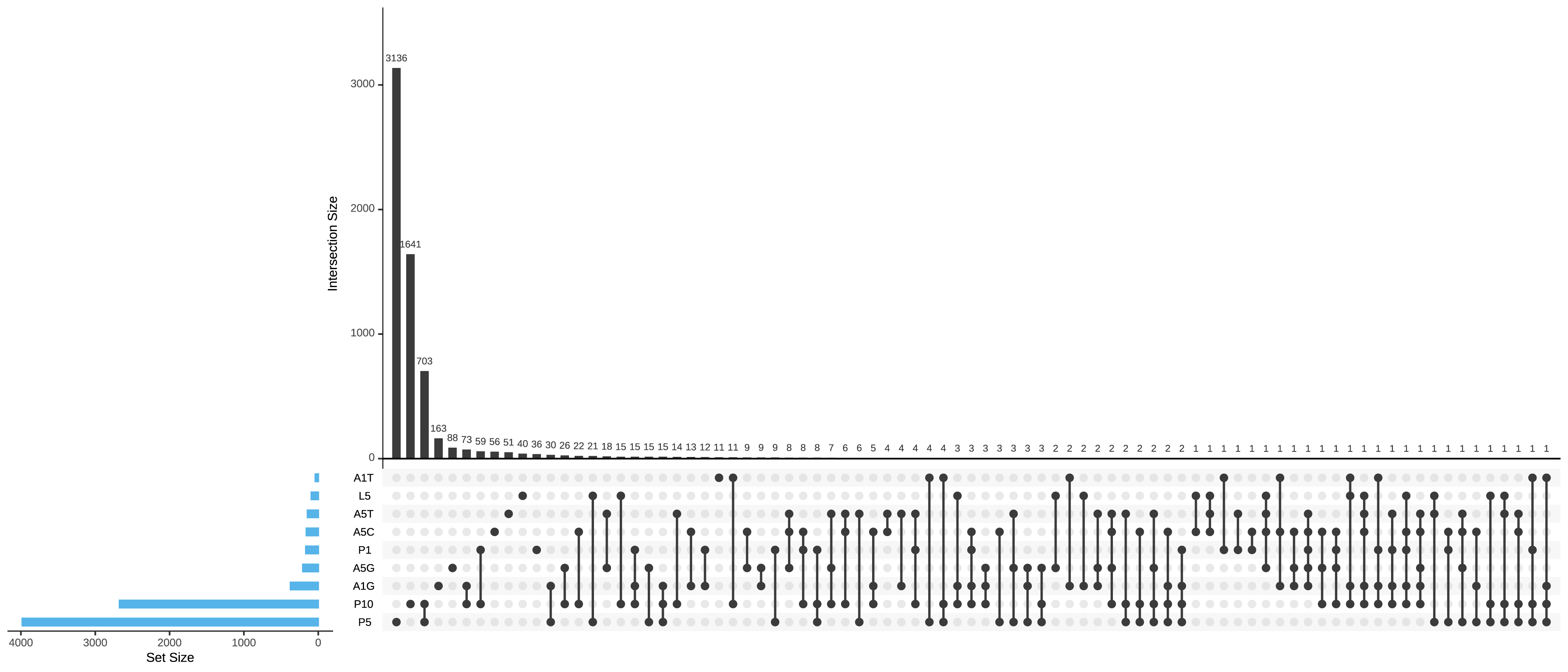

Supplement: Supplementary file 1 [file ijms-25-04906-s001.zip › Supplementary materials/Supplementary figures/Figure S2.tif]

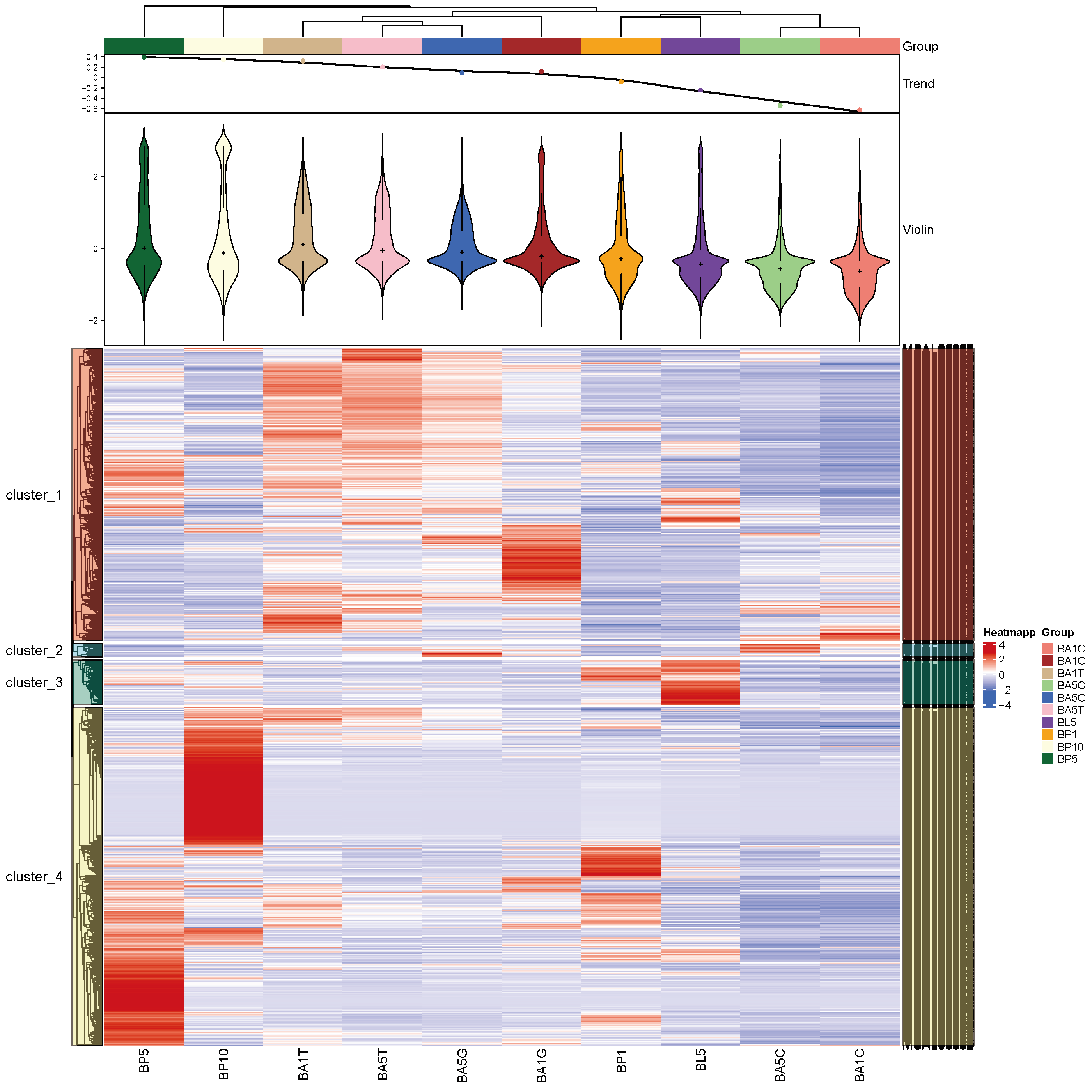

Supplement: Supplementary file 1 [file ijms-25-04906-s001.zip › Supplementary materials/Supplementary figures/Figure S3.tiff]

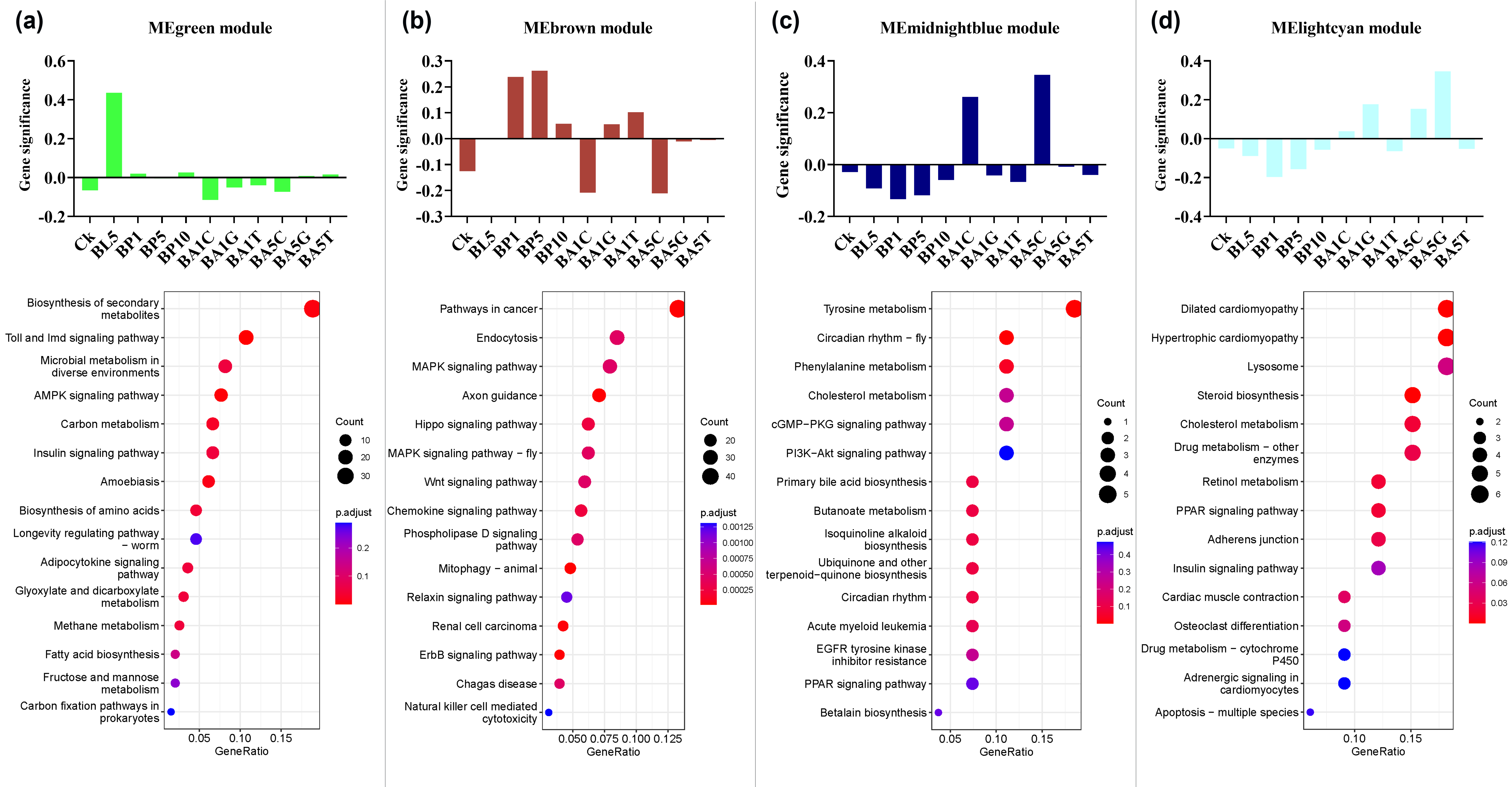

Supplement: Supplementary file 1 [file ijms-25-04906-s001.zip › Supplementary materials/Supplementary figures/Figure S4.tif]

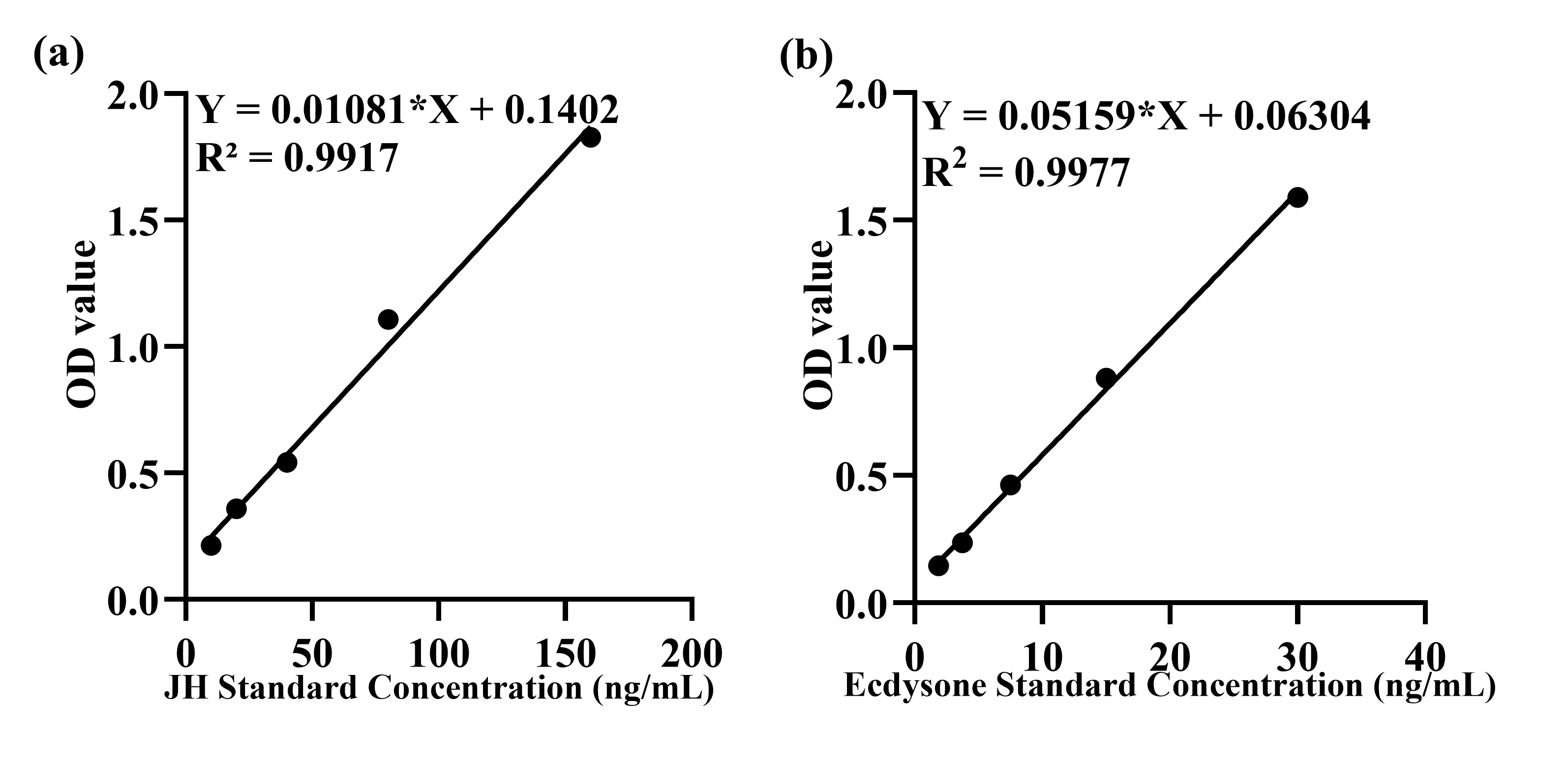

Supplement: Supplementary file 1 [file ijms-25-04906-s001.zip › Supplementary materials/Supplementary figures/Figure S5.tif]

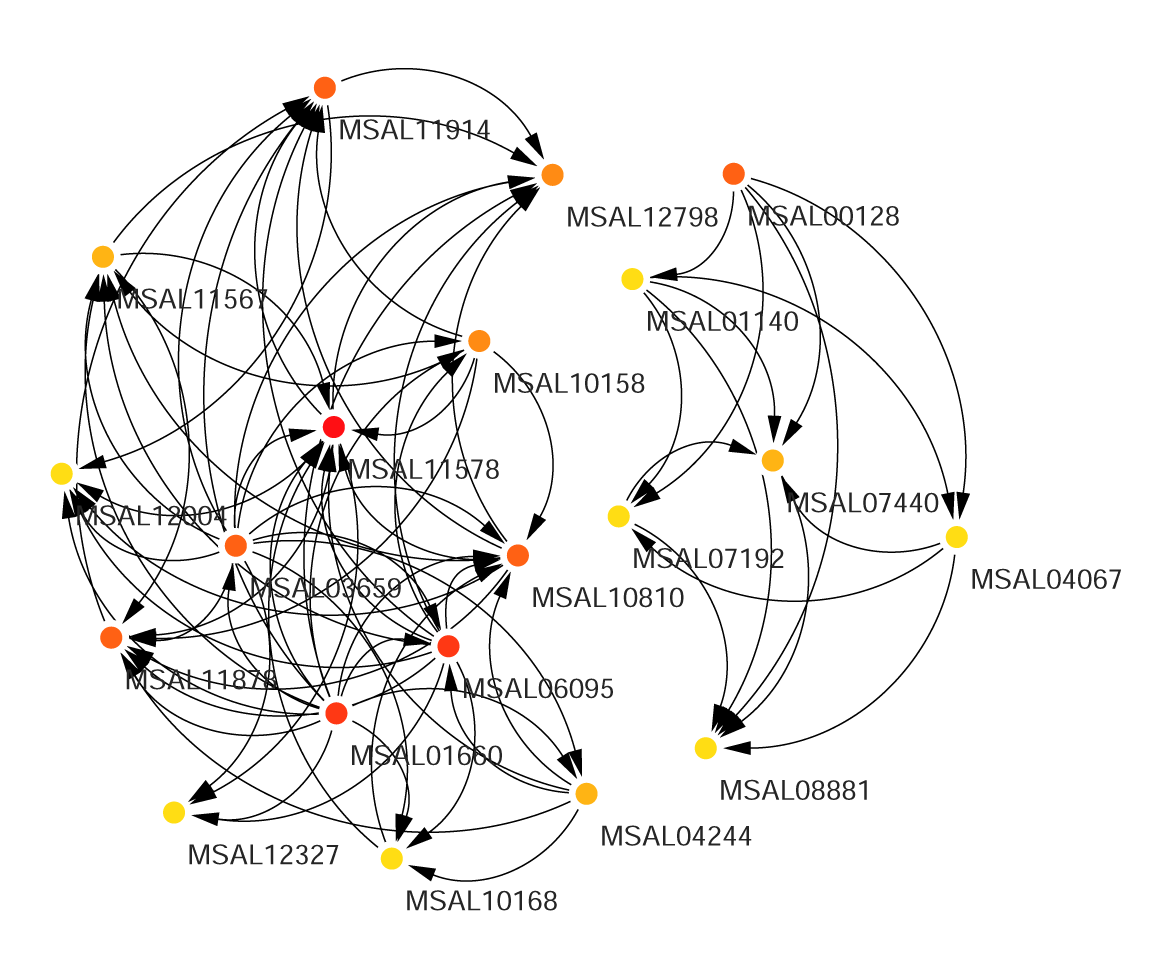

Supplement: Supplementary file 1 [file ijms-25-04906-s001.zip › Supplementary materials/Supplementary figures/Figure S6.tif]

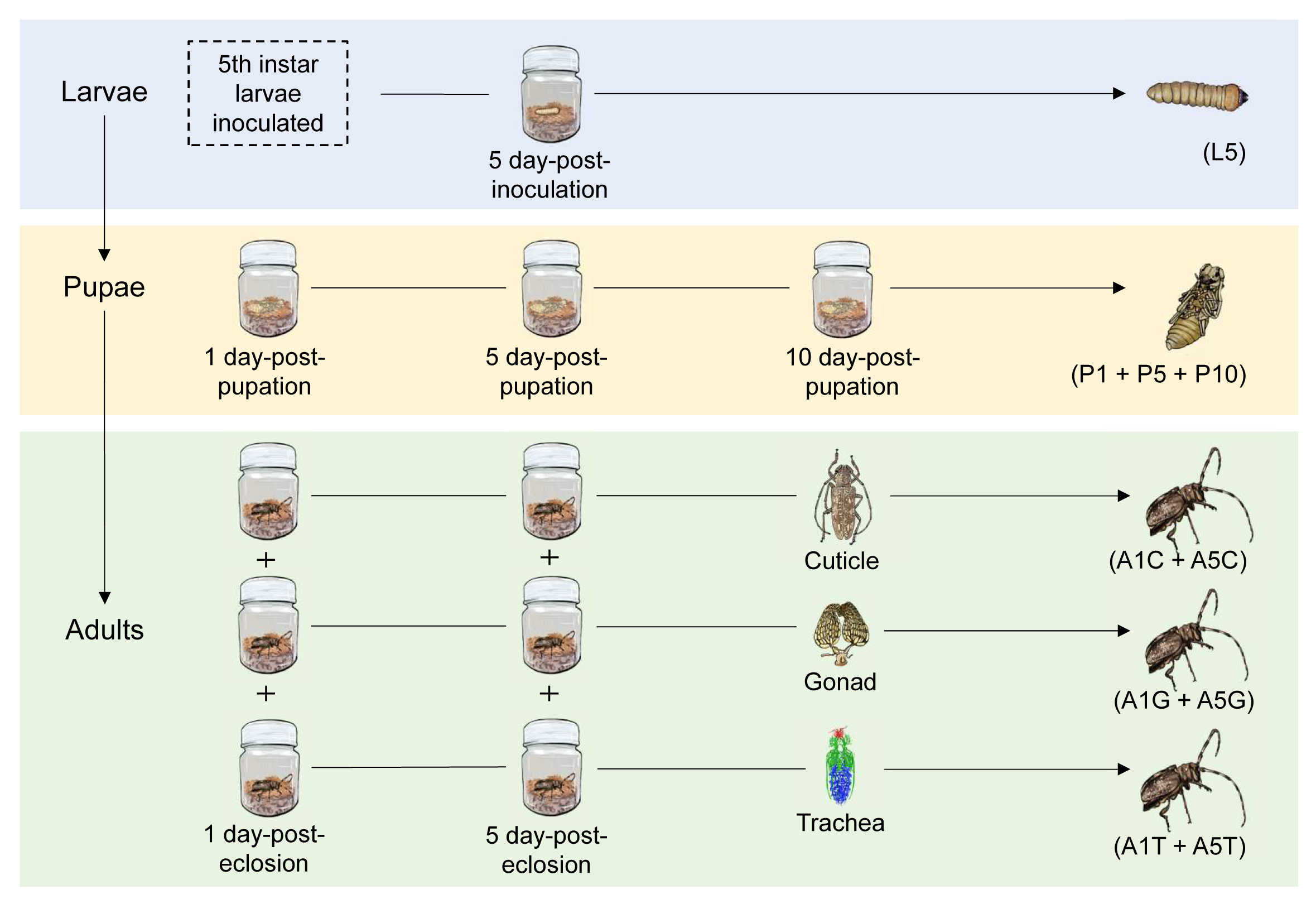

Supplement: Supplementary file 1 [file ijms-25-04906-s001.zip › Supplementary materials/Supplementary figures/Figure S7.tif]

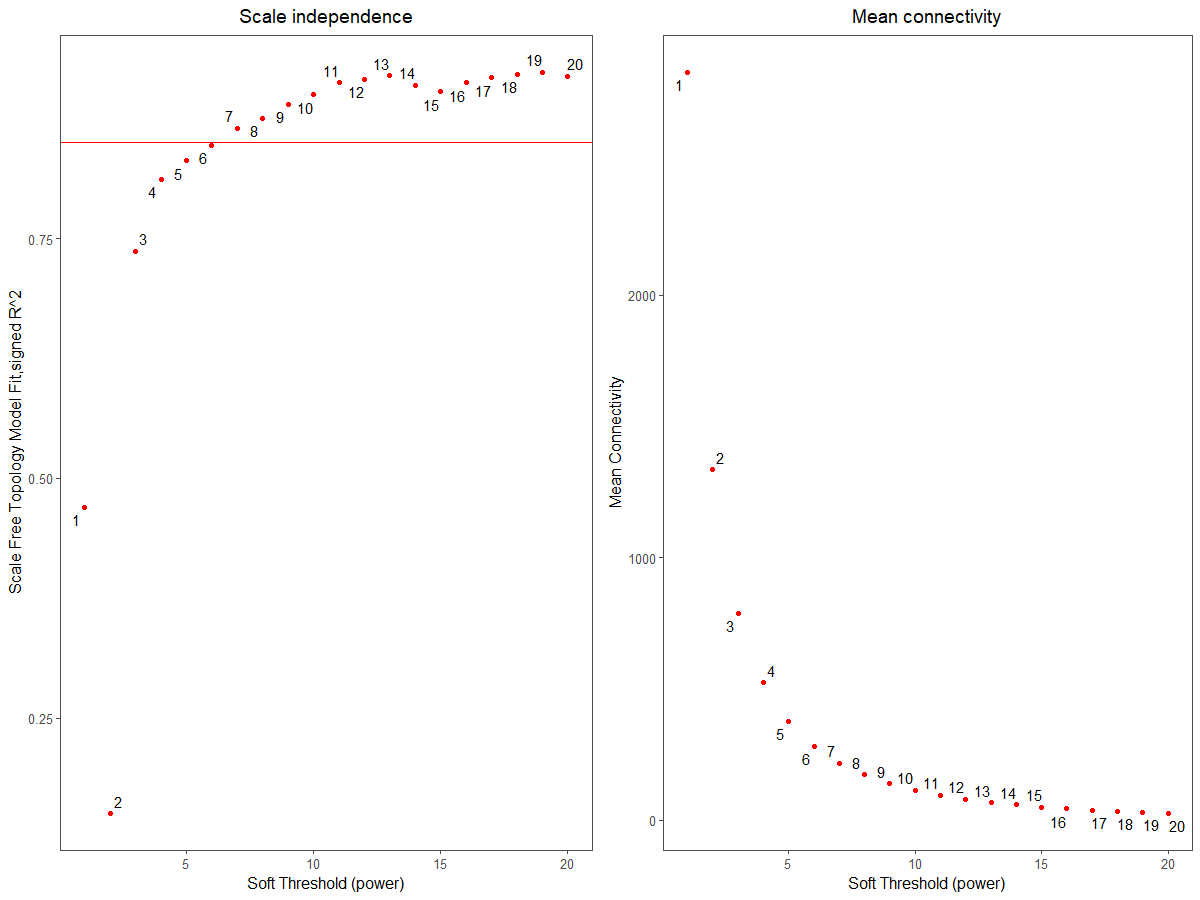

Supplement: Supplementary file 1 [file ijms-25-04906-s001.zip › Supplementary materials/Supplementary figures/Figure S8.png]
